# Native Rhizospheric and Endophytic Fungi as Sustainable Sources of Plant Growth Promoting Traits to Improve Wheat Growth under Low Nitrogen Input

**DOI:** 10.3390/jof8020094

**Published:** 2022-01-19

**Authors:** Akram H. Mohamed, Fayrouz H. Abd El-Megeed, Naziha M. Hassanein, Sameh H. Youseif, Peter F. Farag, Saleh A. Saleh, Basel A. Abdel-Wahab, Amnah Mohammed Alsuhaibani, Yosra A. Helmy, Ahmed M. Abdel-Azeem

**Affiliations:** 1 Department of Microbial Genetic Resources, National Gene Bank, Agricultural Research Center (ARC), Giza 12619, Egypt; akramhassan8787@gmail.com (A.H.M.); fayrouz_1983@hotmail.com (F.H.A.E.-M.); samehheikal@hotmail.com (S.H.Y.); 2 Microbiology Department, Faculty of Science, Ain Shams University, Abbasia 11566, Egypt; naziha_hassanein@sci.asu.edu.eg (N.M.H.); peter_jireo@sci.asu.edu.eg (P.F.F.); 3 Agricultural Microbiology Research Department, Soils, Water and Environment Research Institute, Agricultural Research Center (ARC), Giza 12619, Egypt; sasaleh2006@hotmail.com; 4 Department of Medical Pharmacology, College of Medicine, Assiut University, Assiut 7111, Egypt; basel_post@msn.com; 5 Department of Pharmacology, College of Pharmacy, Najran University, P.O. Box 1988, Najran 55461, Saudi Arabia; 6 Department of Physical Sport Science, College of Education, Princess Nourah bint Abdulrahman University, P.O. Box 84428, Riyadh 11671, Saudi Arabia; amalsuhaibani@pnu.edu.sa; 7 Department of Animal Hygiene, Zoonoses and Animal Ethology, Faculty of Veterinary Medicine, Suez Canal University, Ismailia 41522, Egypt; helmy.6@osu.edu; 8 Botany and Microbiology Department, Faculty of Science, Suez Canal University, Ismailia 41522, Egypt

**Keywords:** IAA, phosphorus, fungi, endophytes, wild plants, fertilization, crop protection, rhizosphere

## Abstract

Wheat crops require effective nitrogen fertilization to produce high yields. Only half of chemical N_2_ fertilizers are absorbed into plants while the rest remains in the soil, causing environmental problems. Fungi could maximize nitrogen absorption, and from an environmental and biodiversity point of view, there is an urgent necessity for bioprospecting native fungi associated with wild plants growing in harsh environments, e.g., St. Katherine Protectorate (SKP) in the arid Sinai. Recovered taxa, either endophytic and/or rhizospheric, were screened for their plant growth-promoting (PGP) traits. Eighteen fungal isolates (15 rhizospheric and 3 endophytic) belonging to anamorphic ascomycetes were recovered from 9 different wild plants, and their PGP traits (indole-3-acetic acid [IAA] production, phosphate solubilization, siderophore production, and hydrolytic enzyme production) were measured. Rhizospheric isolate NGB-WS14 (*Chaetosphaeronema achilleae*) produced high levels of IAA (119.1 μg mL^−1^) in the presence of tryptophan, while NGB-WS 8 (*Acrophialophora levis*) produced high IAA levels (42.4 μg mL^−1^) in the absence of tryptophan. The highest phosphate-solubilizing activity (181.9 μg mL^−1^) was recorded by NGB-WFS2 (*Penicillium chrysogenum*). Endophytic isolate NGB-WFE16 (*Fusarium petersiae*) exhibited a high percentage level of Siderophore Unit (96.5% SU). All isolates showed variability in the secretion of extracellular hydrolytic enzymes. Remarkably, all isolates had antagonistic activity (55.6% to 87.3% suppression of pathogen growth) against the pathogenic taxon *Alternaria alternata* (SCUF00001378) in the dual-assay results. Out of the 18 isolates, 4 rhizospheric and 1 endophytic isolate showed significant increases in shoot dry weight and shoot nitrogen and chlorophyll content of wheat plants subjected to low inputs of chemical nitrogen (N) fertilizers (50% reduction) compared with the non-inoculated control in a pot experiment. Potent taxa were subjected to sequencing for molecular confirmation of phenotypic identification. The retrieved ITS sequences in this study have been deposited in GenBank under accession numbers from LC642736 to LC642740. This study considered the first report of endophytic fungi of *Cheilanthes vellea,* a wild plant with PGPF which improves wheat growth. These results recommend using PGPF as inoculants to alleviate low nitrogen fertilization.

## 1. Introduction

Wild medicinal plants are a reservoir of many bioactive compounds that are safe for humans and the environment compared with chemical and synthetic compounds used to treat many diseases [1]. Many reports suggest that wild plants growing in harsh conditions may harbor plant growth-promoting (PGP) rhizobacteria. However, very little is known about the microbiota that colonizes the roots of desert plants [2]. Microorganisms that surround and inhabit wild medicinal plants play an important role in production-specific secondary metabolites [3]. Rhizospheric fungi build up a complex interaction between plants and soil by utilizing nutrients released by a host plant. Fungi play a vital role in the development of sustainable agriculture [4]. Endophytic fungi are considered symbiotic organisms because they colonize healthy tissues of varied plant species asymptomatically [5,6,7,8]. As reported by [9], endophytic fungi could be categorized into two large groups, clavicipitaceous endophytes, which could infect some grasses, and nonclavicipitaceous endophytes.

Plant growth-promoting fungi (PGPFs) can enhance plant growth and crop yields through many traits such as levels of indole-3-acetic acid (IAA), siderophore production, and cellulase and chitinase secretion [10,11,12]. PGPFs that have beneficial effects on plants without causing diseases, such as *Trichoderma*, *Aspergillus,* and *Penicillium,* have been widely used as PGPFs in agriculture [10]. The Sinai Peninsula is an epicenter of wild medicinal plants in the Arabian Desert. Medicinal plants are characterized by biological activity that is beneficial for humans and/or animals. Wild medicinal plants in Sinai attract the attention of many ecologists, taxonomists, and phytochemists owing to their importance as large reservoirs for a diverse microbial community that has a crucial role in alleviating drought stress through increasing biomass production and enhancing plant growth [11,12,13,14]. The PGP activity of wild plants associated with fungi has been previously described in several studies. For instance, [13] isolated the endophytes *Penicillium chrysogenum* and *Penicillium crustosum* from *Teucrium polium* L. in the Saint Katherine Protectorate (SKP) in Egypt and found that they had PGP activity on maize plants.

The wheat crop (*Triticum aestivum* L.) is the most important cereal crop in the world for human food, animal feed, and biofuel security [14,15]. In 2018, wheat production worldwide was estimated at 733.40 million tons. In Egypt, the wheat crop is grown on about 20% of the total cultivated agricultural land, or 1.28 M ha, with a production of 9.00 Mt [16]. One has to consider the expected increase in the need for wheat crop production. It is estimated that by 2050, consumers will require 60% more than today [17]. Wheat crop improvement requires large amounts of nitrogen fertilization for ideal protein content. Cereal plants absorb only about 50% of applied nitrogen. However, applying excessive N fertilizers decreases the percentage of N absorbed by plants, so there is a crucial need for optimizing N uptake by plants [18,19,20]. It is traditionally known that Arbuscular Mycorrhizal Fungi (AMF) play an important role in nitrogen uptake by plants through symbiosis with plant roots [21]. Exploring new fungal species with multi-PGP traits other than AMF from wild plants is necessary.

The main objective of this study concentrates on the isolation, molecular identification, and characterization of potent fungal rhizospheric and endophytic taxa related to some wild and medicinal plants that occur in arid conditions of Saint Katherine Protectorate, Egypt. Our study focuses on investigating fungal endophytes of *Cheilanthes vellea, Conyza stricta*, and *Silene schimperiana* and exploring their role in plant growth-promoting activity. This study represents the first report about endophytic fungi of rare fern *Cheilanthes vellea* and their role in wheat plant growth promotion. More specifically, verification for plant growth-promoting traits of these isolates such as extracellular enzymatic production, antagonistic activity against pathogenic *Alternaria*, IAA, siderophore production, and P solubilization were evaluated to test their influence on the biomass production of wheat plants as an important economical crop under low nitrogen input.

## 2. Materials and Methods

### 2.1. Sample Collection

The SKP is situated in the southern part of Sinai and is a part of the upper Sinai massif (33°550 to 34°300 E and 28°300 to 28°350 N) located at an elevation of 1500 to 2624 m above sea level. Nine wild plants and the rhizospheric soil were collected in sterile plastic bags and then transferred in an ice box to the lab for further work. Samples were collected with the permission of the SKP for scientific purposes, and no endangered species were involved in the study (Table 1). Taxonomic identification of the plants under investigation was conducted by [22] and confirmed in the Cairo University Herbarium (CAI), Botany and Microbiology Department, Faculty of Science, Cairo University by Professor Wafaa Amer.

### 2.2. Isolation of Rhizospheric and Endophytic Fungi

For the isolation of rhizospheric fungi, the dilution plate technique according to [23] was consulted. Czapek’s yeast extract agar (CYA) and potato dextrose agar (PDA) were used as isolation media and supplemented with Rose Bengal (1/15,000) as a bacteriostatic agent and chloramphenicol (50 ppm) for the suppression of bacterial growth [24]. The plates were incubated at 25 ± 2 °C for 7 days, and thereafter the developing colonies were identified and counted.

Pieces of plant parts (roots, stems, and leaves) were surface sterilized and cut according to the method by [25] before being plated on appropriate isolation media (4 pieces 5 mm^2^/plate). To check the sterilization efficiency, the last washing water was spread onto PDA plates, and the growth, if any appeared, was compared with the incubated plates with surface-sterilized plant pieces. For primary isolation, the same media were used in the isolation of rhizospheric fungi.

### 2.3. Phenotypic Identification

Taxonomic identification of isolated fungi used the phenotypic approach down to the species level on standard media based on the following identification keys: for *Penicillium* [26]; for *Aspergillus* [27,28,29]; for dematiaceous hyphomycetes [30,31]; for *Fusarium* [32]; for miscellaneous fungi [33], and for Ascomycetes [34]. The names of authors of fungal taxa are abbreviated according to [35]. The systematic arrangement in the present list follows the latest system of classification appearing in the 10th edition of Ainsworth and Bisby’s *Dictionary of the Fungi* [36]. Name corrections, authorities, and taxonomic assignments of all taxa reported in this work were checked against the Index Fungorum database (www.indexfungorum.org (accessed on 22 September 2021)).

### 2.4. Screening of PGP Trails

#### 2.4.1. Production of Indole-Acetic Acid

Malt extract broth (2%) supplemented with 0.1% (*w/v*) L-tryptophan (pH 5.5) was used for IAA production in submerged conditions [37]. The inoculated flasks were incubated on a rotary shaker at 30 °C and 150 rpm for 10 days. After the incubation period, the culture media were centrifuged (4000 rpm for 10 min), and 1 mL of supernatant was combined with 2 mL of Salkowski’s reagent and incubated for 30 min at room temperature. The production of IAA was determined by colorimetric measurement at 530 nm using a spectrophotometer (Thermo Scientific Evolution 100, Thermo Fisher Scientific, Waltham, MA, USA) as described by [38].

#### 2.4.2. Phosphate Solubilization

The quantitative estimation of tri-calcium phosphate (TCP) solubilization by each fungal isolate was done in Pikovskaya’s liquid broth medium [39]. TCP was added to each flask (*w/v*) after incubation for 10 days at 180 rpm. The supernatant of the fungal filtrate was obtained by centrifugation (10,000× *g* for 10 min) according to the method of [40]. Phosphate solubilization was quantified by the phosphomolybdate blue color method using a standard graph of monopotassium phosphate (KH_2_PO_4_) and expressed in parts per million (ppm) at 600 nm using a spectrophotometer (Thermo Scientific™ Evolution 100). pH variations of the medium were also monitored.

#### 2.4.3. Antagonistic Activity against *Alternaria alternata* In Vitro

Fungal isolates were subjected to screening for antagonistic activity against the wheat pathogenic isolate *Alternaria alternata* (SCUF00001378) via a dual-culture technique [41] based on the percentage of inhibition of radial growth (PIRG).

Antagonistic activity of the tested fungal isolates was assessed after 7 days of incubation by measuring the radius of the *A. alternata* colony using the following formula:

Percentage (%) of inhibition of radial growth (PIRG) = (R_1_ − R_2_/R_1_) × 100

where R_1_ is the radial growth of the fungal colony on the control plate and R_2_ is the radial growth of the fungal colony in the dual culture.

#### 2.4.4. Production of Siderophore

Tested isolates were checked for their siderophore-producing ability by the universal chrome azurol S (CAS) assay [42]. The quantitative estimation of siderophore for fungal isolates was performed using 96-well plates. The optical density was 630 nm as measured by a microplate reader (Infinite 200 Pro, Life Sciences/Tecan, Mannedorf, Switzerland) according to [43]. Siderophore produced by strains was measured as the percentage of siderophore unit (% SU), which was calculated according to the following formula [44]:

Percentage of siderophore unit (% SU) ꞊ (A_r_ − A_S_)/A_r_ × 100 (1)

where A_r_ is the absorbance of reference (CAS solution and uninoculated broth) and A_S_ is the absorbance of the sample (CAS solution and cell-free supernatant of sample).

#### 2.4.5. Extracellular Enzymes

The ability of fungal isolates to produce hydrolytic enzymes was screened on basal medium [45] supplemented with (1% *w/v*) different sole carbon sources (carboxymethyl cellulose [CMC], oat pelt xylan, and pectin of citrus peel, as well as colloidal chitin) for the testing of the production of cellulase, xylanase, pectinase, and chitinase, respectively. After incubation for 10 days on a specific medium, the plates were flooded with Gram’s iodine for 5 min. Plates were then observed for halozones [46,47,48,49]. The clear zones around colonies indicated qualitative enzyme activity [43,44,45]. The enzyme index (EI) was calculated according to the following formula:

EI = Diameter of hydrolysis zone (cm)/Diameter of colony (cm)(2)

### 2.5. Application of Fungal Isolates as PGP agents for Wheat Plants

#### 2.5.1. Vigor of Wheat Plant Seedlings

The seedling vigor was measured according to [50]. Both a fungal culture and a spore suspension of the fungal isolates were tested to determine their effects on wheat seedling germination and scaled according to the vigor index [51]. Fifty surface-sterilized wheat grains (Misr1) were soaked in 10 mL of fungal spore suspension (1.0 × 10^8^ spores/mL) for each isolate and kept at 25 ± 2 °C in a rotary shaker for 6 h to ensure uniform coating. To study the effect of the culture filtrate on the germination of wheat grains, 10 grains per Petri dish were maintained. Grains were soaked in each fungal filtrate for 12 h. Grains were placed into sterilized Petri dishes containing sterilized cotton layers [52]. Each treatment was done in three replicates, and the control experiment was treated and maintained with sterilized culture media without a fungal inoculum as the control with the same number of replicates. After 7 days of incubation at 25 °C in dark conditions, percentages of germination, as well as the plumule and radicle lengths of the seedlings, were determined. The vigor index was calculated using the following mathematical equation:

Vigor index = {Length of plumule (cm) + Length of radicle (cm)} × Percentage of germination

#### 2.5.2. Pot Experiment

Measurements of grain germination and the vigor of seedlings were conducted. Out of 18 fungal isolates, 16 were tested to evaluate their potential for improving the growth of wheat plants under low nitrogen (N) inputs. Low-fertility sandy soil was collected from the Ismailia Agricultural Research Station, Agricultural Research Center (30°36′56.94″ N, 32°14′39.68″ E). Pots of 13 cm in diameter were filled with 2.5 kg of sandy soil. Ten grains of wheat variety Misr1 were cultivated per pot. All inoculated pots received 50% of the recommended N dose of ammonium sulfate (20.5% N) at a rate of 0.73 g/pot (144 kg N/ha), as recommended by the Ministry of Agriculture and Land Reclamation of Egypt (www.caaeeg.com (accessed on 15 November 2019)). Control treatments were 50% N as the recommended dose and the full recommended dose of 100% N without microbial inoculation. Each pot was inoculated with 5 mL of the spore suspension of the fungal isolates (10^6^ spores/mL) at planting. All treatments received the recommended dose of super phosphate (12.5% P_2_O_5_) and potassium sulfate (48.5% K_2_SO_4_) at the rate of 0.5 g/pot (480 kg/ha) and 0.25 g/pot (240 kg/ha), respectively. Phosphate fertilizer was applied before planting, and the N and potassium (K) fertilizers were split into 3 doses (10, 20, and 30 days after planting). After 10 days, the plants were thinned to 6 plants/pot. All treatments were irrigated with tap water and arranged in a completely randomized block design with three replicates. The plants were uprooted after 50 days of cultivation. The plant height, fresh weight of shoots and roots, dry weight of shoots and roots, and shoot N content, in addition to the photosynthetic pigments of the inoculated plants and the uninoculated controls, were tabulated and subjected to data analysis for the determination of the isolate that improved wheat growth [53].

### 2.6. Molecular Confirmation and Phylogenetic Analysis of Most Potent PGPF Isolates

The fungal culture was centrifuged, and the pellet was ground using a plastic rod. Then, genomic DNA was extracted using the DNeasy Plant Mini Kit (Qiagen, Hilden, Germany). Polymerase chain reaction (PCR) amplification of the ITS rRNA region was done using ITS1 (5′-TCCGTAGGTGAACCTGCGG-3′) and ITS4 (5′-TCCTCCGCTTATTGATATGC-3′) primers, as described by [54]. PCR was performed using the standard reaction mixture (50 µL): 1× PCR buffer, 1.5 mM of MgCl_2_, 200 mM of each dNTP, 15 pmol of each primer, 1U of Taq polymerase enzyme, and 50 ng of the DNA template. PCR was performed as follows. Primary denaturation was done for 3 min at 94 °C; 30 cycles of denaturation were done at 94 °C for 30 s; annealing was done at 58 °C for 30 s; extension was done at 72 °C for 90 s; final extension was done at 72 °C for 10 min. The PCR products were detected on 1.5% agarose gel electrophoresis and then were purified using the PureLink PCR Purification Kit (Thermo Fisher Scientific). PCR products were sequenced at Macrogen, Inc. (Seoul, Korea) and searched for similar sequences in their rRNA/ITS databases with GenBank (https://blast.ncbi.nlm.nih.gov/, accessed on 14 July 2021) using the BLAST function. The retrieved ITS sequences in this study have been deposited in GenBank under accession numbers from LC642736 to LC642740. The sequences were aligned using Clustal W version 1.8 [55] and subjected to phylogenetic analyses. The phylogenetic tree was constructed using the maximum likelihood [56] in MEGA X version 10 [57] using the Tamura-Nei model. Bootstrap support for each node was evaluated with 1000 replicates. The average nucleotide identity was calculated between isolated fungi and closely related reference strains using MEGA X software.

### 2.7. Statistical Analysis

The effect of fungal inoculation was analyzed using the one-way ANOVA performed with SPSS 20.0 Statistics (IBM SPSS, Somers, NY, USA). All results were expressed as the mean ± standard deviation (SD). The significance of differences within treatments was separated using Duncan’s multiple range tests at a probability level of 0.05.

## 3. Results

### 3.1. Isolation and Identification of Rhizospheric and Endophytic Fungi

A total of 18 fungal isolates (15 rhizospheric, 3 endophytic) were isolated from 9 different wild medicinal plants belonging to 6 different plant families from 7 different locations in the SKP (Table 2).

The rhizospheric soil of wild plants *Diplotaxis harra* (Forssk.) Boiss and *Peganum harmala* L were the richest in habitat with the highest number of culturable fungal isolates. The isolated endophytic fungi were recovered from *Cheilanthes vellea* (Aiton) F. Muell, *Conyza stricta* Willd., and *Silene schimperiana* Boiss. Plants.

### 3.2. Screening the PGP Traits of Fungal Isolates

#### 3.2.1. IAA Production

The fungal isolates were tested for their PGP traits, including IAA and siderophore production, in addition to their phosphate solubilization efficiency.

**Figure 1:**
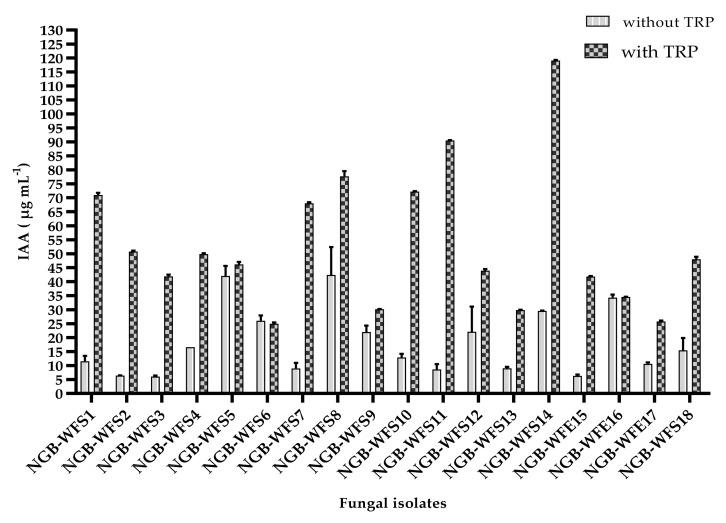
IAA production by tested fungal isolates with medium supplemented by tryptophan and without tryptophan. All values are mean with SEM.

The findings (Figure 1) showed that all fungal isolates produced IAA, with or without tryptophan. IAA production ranged from 25.8 µg mL^−1^ by the endophytic isolate NGB-WFE17 (*Alternaria botrytis)* to 119.1 µg mL^−1^ by NGB-WFS14 (*Chaetosphaeronema achilleae*).

In the absence of tryptophan, isolate NGB-WFS5 (*Aspergillus fumigatiaffinis*) from the rhizosphere of *Sonchus oleraceus* produced the highest amount of IAA, 42.0 µg mL^−1^. Those may be due to the root exudates of *sonchus olearceous,* which could support the precursor for IAA biosynthesis regardless of its presence in the production medium. The lowest amount of IAA (6.1 µg mL^−1^) was produced by NGB-WFS3 (*Penicillium chrysogenum*) from the rhizosphere of *Thymus bovei.*

As observed (Figure 1), isolating NGB-WFS6 (*Alternaria alternata)* from the rhizosphere of *Peganum harmala* could produce similar IAA levels (24.9 and 26.0 µg mL^−1^) in the presence and absence of tryptophan, respectively. In the same manner, endophyte NGB-WFE16 (*Fusarium petersiae*) of *Conyza stricta* could produce equal IAA levels (34.3 µg mL^−1^), regardless of the presence or absence of tryptophan. As a general observation from previous results, IAA production could be supported by the host/source of isolation since rhizospheric isolates (NGB-WFS4, NGB-WFS 7, and NGB-WFS 14) which isolated from different plant rhizospheres (*Marrubium alysson, Peganum harmala,* and *Diplotaxis harra),* respectively, were identified as *Chaetosphaeronema achilleae* showed different IAA patterns in presence or absence of precursor.

#### 3.2.2. Phosphate Solubilization Efficiency

**Figure 2:**
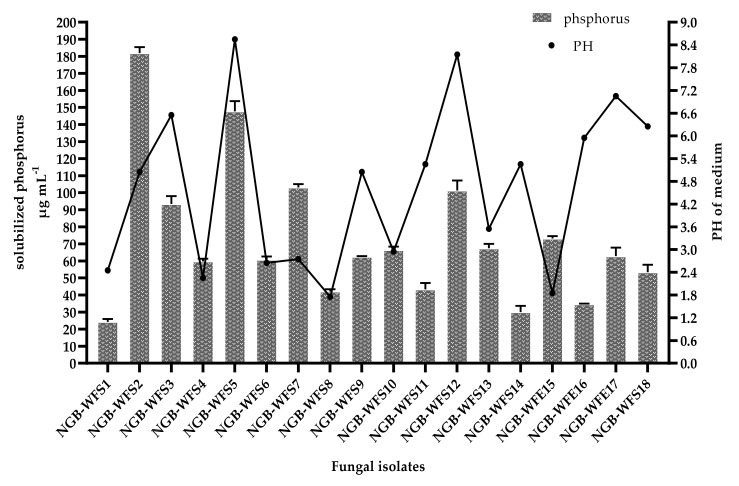
Solubilized P concentrations and corresponding PH of PVK broth inoculated with tested fungal isolates after 10 days of incubation.

As shown in Figure 2, the maximum amount of solubilized phosphorus was 181.9 µg mL^−1^, obtained by the NGB-WFS2 (*Penicillium chrysogenum*) isolate from the *Thymus bovei* rhizosphere, while the lowest amount of solubilized phosphorus was 24.2 µg mL^−1^, obtained by the NGB-WFS1 (*Botryotrichum atrogriseum*) isolate from the rhizosphere of *Cleome droserifolia*.

As indicated in Figure 2, some of the isolates were able to decrease the pH of the Pikovskaya’s medium, and this indicated a high amount of solubilized phosphorus. This was observed in the case of NGB-WFS7 (*Chaetosphaeronema achilleae)*, with 103.1 µg mL^−1^ of solubilized phosphorus with an acidic pH of 2.8.

Interestingly, both different rhizospheric isolates NGB-WFS5 (*Aspergillus fumigatiaffinis*) and NGB-WFS 12 (*Penicillium chrysogenum*), could solubilize phosphorus by 147.69 and 101.32 µg mL^−1^, respectively, with alkaline PH 8, from the rhizosphere of the same plant (*sonchus olearceous*), this rise in PH may be due to production of alkaline compounds or due to rapid consumption of organic acids produced in the medium.

On the other hand, rhizospheric isolates exhibited higher solubilized phosphorus than endophytic isolates, as shown in Figure 2. This could be explained due to isolate habitat which supports its ability for phosphate solubilization.

#### 3.2.3. Antagonistic Activity and Siderophore Production

**Figure 3:**
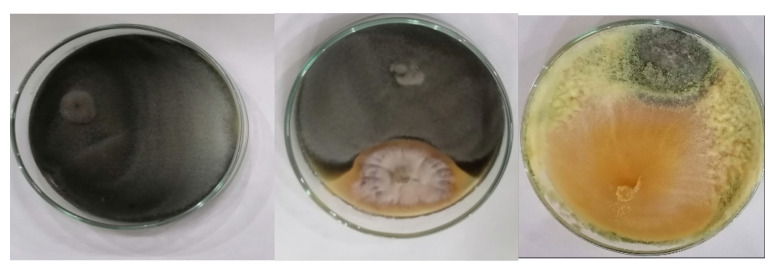
Antagonistic effect of tested fungal isolates against phytopathogen *Alternaria alternate* in dual culture assay (lower side of petri dish fungal isolates NGB-WFS 14 *Chaetosphaeronema achilleae*, NGB-WFS18-*Trichoderma atroviride*).

As Table 3 indicates, all fungal isolates had antagonistic activity of more than 50% against the *A. alternata* pathogen. The highest antagonistic activity, 87.3%, was recorded for NGB-WFS18 (*Trichoderma atroviride*), and the lowest antagonistic activity, 52.4%, was recorded for NGB-WFS14 *Chaetosphaeronema achilleae* (Figure 3). For the siderophore produced by the fungal isolates, Table 3 shows that a high percentage of siderophore units (96.5% and 75.4% SU) were produced by the endophytic isolates NGB-WFE16 *(Fusarium petersiae)* and NGB-WFE15 (*Penicillium chrysogenum)*, endophytes of *C. stricta* and *C. vellea,* respectively, whereas NGB-WFS8 (*Acrophialophora levis*) did not show any siderophore production. As a general observation, as phytopathogen growth inhibition correlated with production of siderophores, not all efficient antagonistic isolates were able to produce siderophores since isolate NGB-WFS8 (*Acrophialophora levis*) showed 77% suppression of phytopathogen *A. alternata* and no siderophores could be detected. This could be explained as pathogen suppression and could be achieved by many other mechanisms like bioactive and volatile organic compounds production.

#### 3.2.4. Extracellular Enzymes

**Figure 4:**
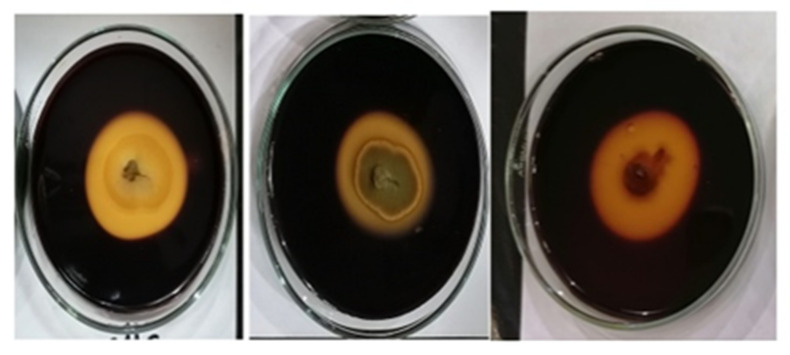
Extracellular enzyme production on the agar plate by fungal isolates showed a clear zone on specific medium stained by iodine. From left to right, NGB-WFS12 on CMC, NGB-WFS12 (*Penicillium chrysogenum*) on xylan, and NGB-WFS11 (*Geotrichum* sp.) on chitin amended medium.

Eighteen fungal isolates were screened for cellulase, pectinase, xylanase, and chitinase activity on a basal salt medium amended with different carbon sources (CMC, pectin, xylan, and colloidal chitin) to test for hydrolytic enzyme production in the isolates. Fungal isolates with high enzymatic activity (EI) values were considered to be potential cellulase, pectinase, xylanase, and chitinase producers, with the presence of a halo colorless zone indicating enzyme production (Figure 4).

All 18 fungal isolates were positive for cellulase and chitinase enzymes, whereas the NGB-WFS6 (*Alternaria alternata)* isolate was negative for both pectinase and xylanase activity.

On the other hand, the NGB-WFS4 (*Chaetosphaeronema achilleae*) and NGB-WFS8 (*Acrophialophora levis*) isolates were negative for xylanase activity. The highest EI (2.0) of cellulase was observed with NGB-WFS2 (*Penicillium chrysogenum*), whereas NGB-WFS1 (*Botryotrichum atrogriseum*) and NGB-WFS11 (*Geotrichum* sp.) showed the highest activity of pectinase with an EI of 1.8, as indicated in Table 4.

The endophytic isolate NGB-WFE16 (*Fusarium petersiae*) exhibited the highest xylanase activity, with an EI of 2.1. The highest chitinase activity, with an EI of 3.9, was measured for the NGB-WFS10 (Sterile mycelium) isolate. From the above results, it can be seen that rhizospheric isolates produced higher levels of chitinase enzyme than obtained for the endophytic isolates. On the other hand, endophytic isolates produced higher levels of xylanase enzyme compared with rhizospheric isolates.

### 3.3. Application of Fungal Isolates as PGP Agents for Wheat Plant

#### 3.3.1. Grain Germination and Seedling Vigor Test

The ability of PGPFs to enhance grain germination and the vigor of the seedling index was evaluated using either spore suspension or a filtrate of a fungal isolate. This experiment was set up to determine the potentiality of either spore suspension and/or culture filtrate. The filtrate under investigation could contain auxins, siderophores, and organic acids, which directly enhance grain germination rapidly until fungal spores germinate and proceed their symbiotic effect on wheat plants in pot trials. On the other hand, spore suspension may infect wheat grains and inhibit their germination. So, it is necessary to test the effect of both fungal filtrate and spore suspension in vitro prior to the pot experiment.

Results (Table 5) revealed that the maximum significance of grain germination percentage and seedling vigor index was 96.5% and 1767, respectively, which were recorded by the filtrate of the NGB-WFE16 (*Fusarium petersiae*). The spore suspension of the NGB-WFS11 (*Geotrichum* sp.) showed the maximum significant rate of grain germination percentage and vigor seedling index as 90.5% and 1533, respectively, among all of the spore suspensions under investigation. The spore suspensions of both NGB-WFS8 (*Acrophialophora levis*) and NGB-WFS14 (*Chaetosphaeronema achilleae*) inhibited the germination of wheat grains. Our results recommended applying both spore suspension and fungal filtrate to enhance the germination and vigor of wheat grains.

**Figure 5:**
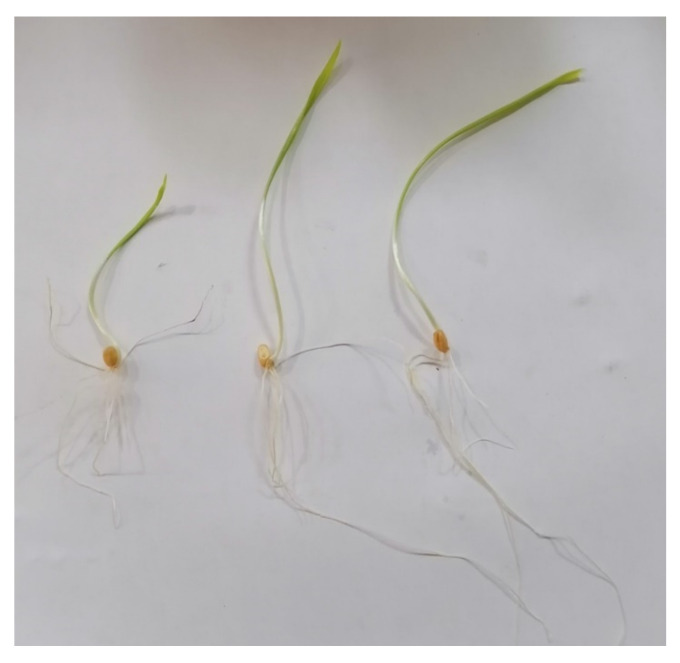
Effect of culture filtrate and spore suspension of fungal isolates on germination and seedling vigor of wheat grains. From left to right, control, wheat grains + culture filtrate of NGB-WFE16 (*Fusarium petersiae*) isolates and wheat grains + spore suspension of NGB-WFS11 (*Geotrichum* sp.) isolates.

The treatment of wheat grains with the most potent fungal isolates enhanced the germination percentage and seedling vigor of the grains, as indicated in Figure 5.

#### 3.3.2. Pot Experiment

**Figure 6:**
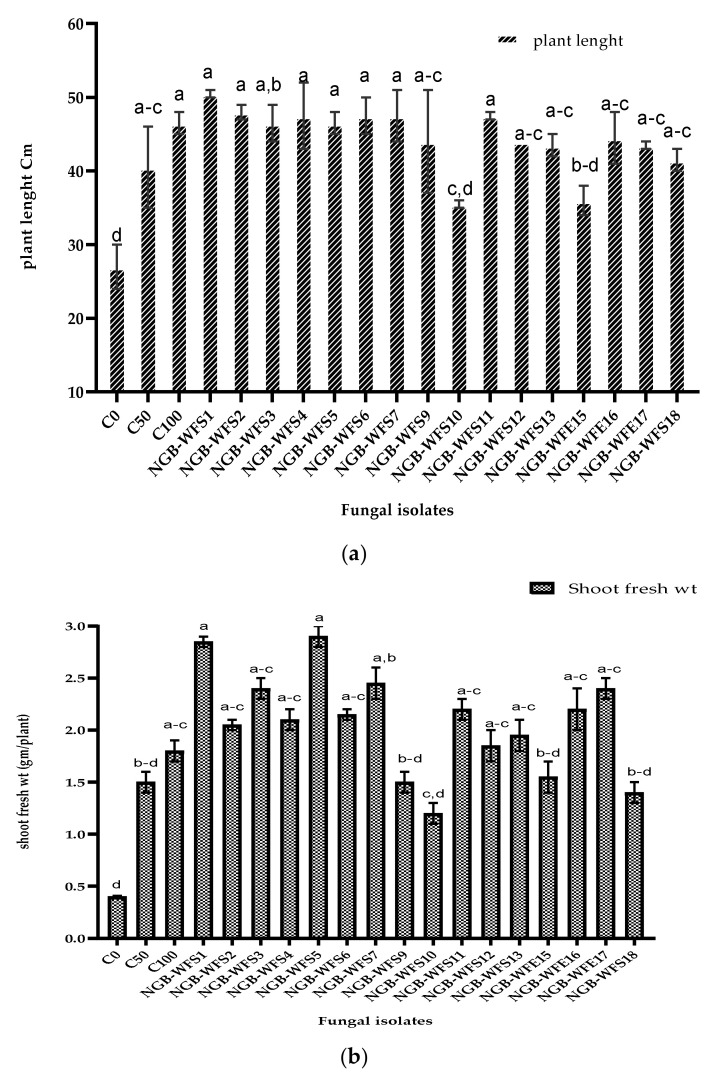

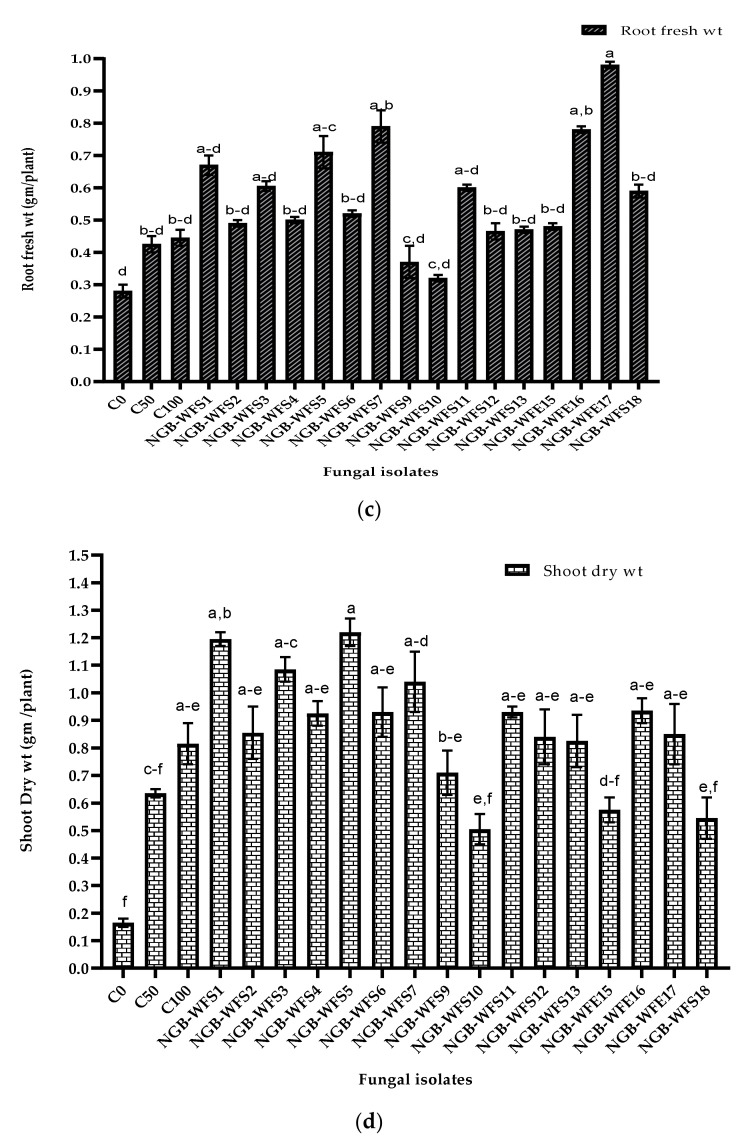

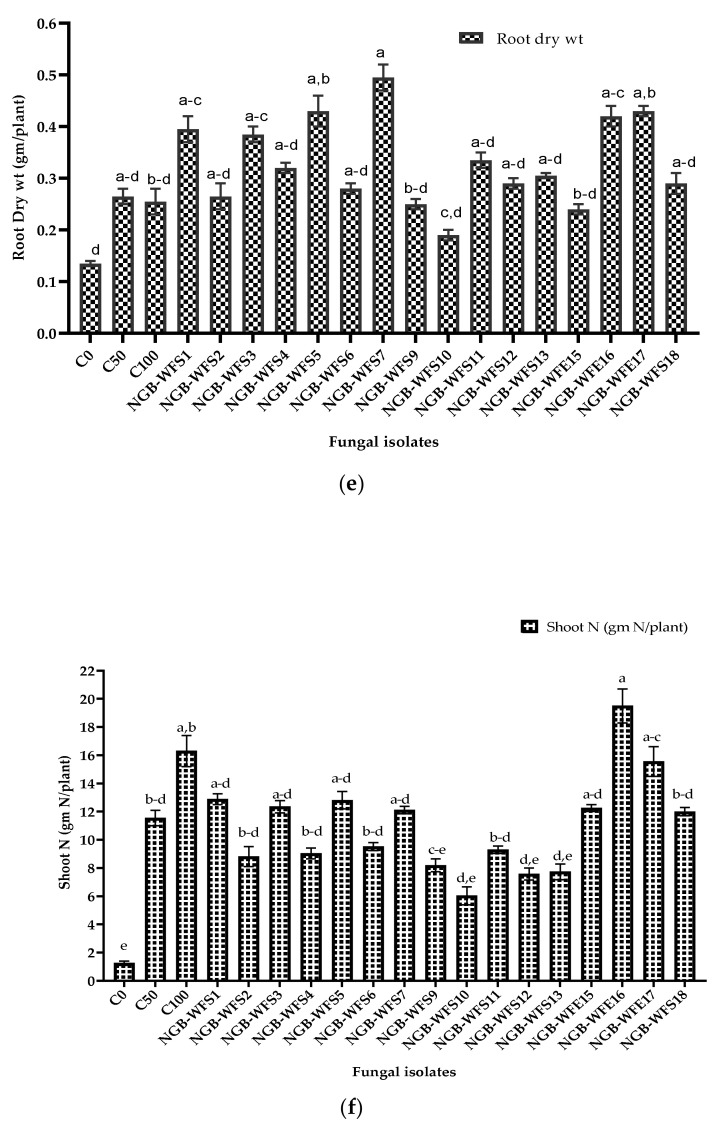
Effects of plant growth-promoting fungi on (**a**) plant height, (**b**) shoot fresh, (**c**) root fresh biomass, (**d**) shoot dry biomass, (**e**) root dry biomass, and (**f**) shoot nitrogen content of wheat in a pot trial. Data are present as a mean of three replicates. Bars sharing different letter(s) are statistically different according to Duncan’s multiple range (*p* ≤ 0.05).

All fungal isolates were tested for their ability to enhance wheat (Misr 1) plant growth under a pot experiment filled with non-sterilized low fertile sandy soil (Table 6). Many plant growth parameters were investigated (i.e., plant height, fresh biomass, dry biomass of shoots and roots, and shoot nitrogen content per plant, as well as photosynthetic pigments of shoots). Results (Figure 6a) showed no significant difference in plant height between inoculated plants and un-inoculated controls. Regarding the shoot fresh weight (Figure 6b), NGB-WFS1 (*Botryotrichum atrogriseum*) and NGB-WFS5 (*Aspergillus fumigatiaffinis)* showed the highest shoot fresh weight by 2.9 g/plant; this was significantly different from the controls, at 50% and 100% N, which had findings of 1.5 g and 1.8 g per plant, respectively, this could be explained as both rhizospheric isolates NGB-WFS1 and NGB-WFS5 could produce high levels of IAA in addition to considerable amounts of solubilized phosphorus which contribute in the enhancement of shoot fresh weight compared to un-inoculated controls.

In the case of the root fresh weight (Figure 6c), NGB-WFE17 (*Alternaria botrytis*) had the highest root fresh weight by 0.985 g/plant, which was significantly different than for the controls at 50% and 100% N.

Inoculation of wheat plants with PGPFs had a great effect on the dry biomass of shoots. Plants inoculated with NGB-WFS1 (*Botryotrichum atrogris*), NGB-WFS5 (*Aspergillus fumigatiaffinis*), and NGB-WFS7 (*Chaetosphaeronema achilleae*) isolates had the highest shoot dry weights by 1.20, 1.22, and 1.04 g/plant, respectively, and the controls at 50% and 100% N had weights of 0.62 and 0.82 g/plant, respectively (Figure 6d). This increase in plant biomass inoculated with fungal isolates is due to the considerable amount of IAA produced by fungal isolates.

Concerning the root dry weight, NGB-WFS7 (*Chaetosphaeronema achilleae*) had the highest value of 0.495 g/plant, which was a significant increase compared with the control at 50% N, which had a weight of 0.255 g/plant (Figure 6e).

Regarding the nitrogen content of the plant shoots, a significant increase was recorded for the NGB-WFE16 (*Fusarium petersiae*) isolate (19.5 mg N/plant) compared with the control at 50% N (Figure 6f).

After observation of the photosynthetic pigments of growing wheat plants, the results (Table 7) showed that there were no significant differences in either chlorophyll a or chlorophyll b between isolates and controls. Regarding the total chlorophyll content of plants, there was a significant increase in the total chlorophyll recorded for the endophytic fungal isolates NGB-WFE15 (3.1 mg g^−1^) and NGB-WFE16 (3.0 mg g^−1^), as well as the rhizospheric isolate NGB-WFS18 (3.0 mg g^−1^), compared with the 100% N control.

As observed, there was a significant increase in the carotenoids obtained by NGB-WFS18 (0.81 mg g^−1^) compared with both controls.

**Figure 7:**
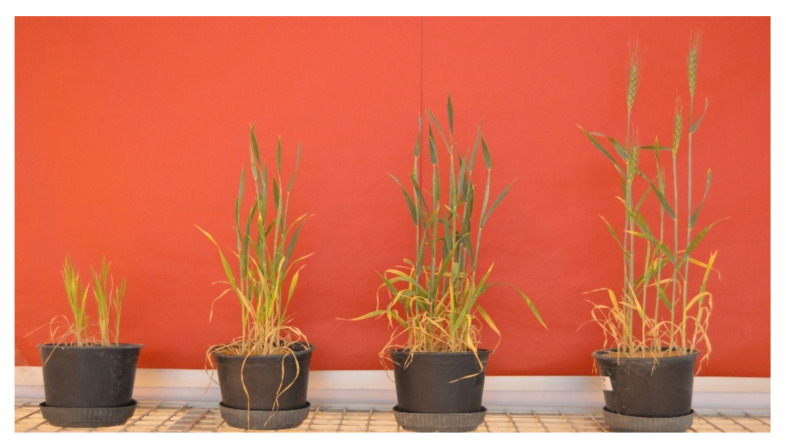
Greenhouse experiment showed the effect of the most potent plant growth-promoting fungal isolates on the growth performance of wheat plants. From right, un-inoculated Control (0 N), control (50 N), control (100 N). Left side, grains inoculated with endophytic fungal isolate (NGB-WFE16) (*Fusarium petersiae*) + 50 N fertilization.

Overall, inoculation of the most promising PGPF isolates enhanced the phenotype and growth parameters in the pot trial, as indicated in Figure 7.

Regarding the total chlorophyll content of plants Table 7, there was a significant increase in the total chlorophyll recorded for the endophytic fungal isolates NGB-WFE15 (3.1 mg g^−1^) and NGB-WFE16 (3.0 mg g^−1^), as well as the rhizospheric isolate NGB-WFS18 (3.0 mg g^−1^), compared with the 100% N control. As observed, there was a significant increase in the carotenoids obtained by NGB-WFS18 (0.81 mg g^−1^) compared with both controls. Overall, inoculation of the most promising PGPF isolates enhanced the phenotype and growth parameters in the pot trial, as indicated in Figure 7.

### 3.4. Molecular Confirmation and Phylogenetic Analysis of Most Potent PGPF Isolates

**Figure 8:**
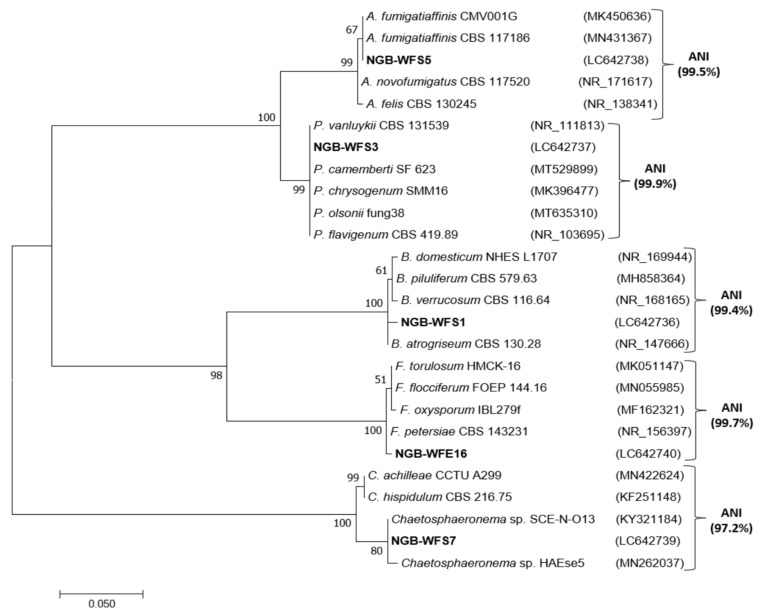
Maximum likelihood (ML) phylogenetic tree based on rDNA ITS sequences of fungal isolates (in bold) and fungal ITS sequences from the GenBank. GenBank accessions are in parentheses. Bootstrap values are indicated for each nod (1000 replicates. A: *Aspergillus*; B: *Botryotrichum*; C: *Chaetosphaeronema*; F: *Fusarium,* and P: *Penicillium*.

Based on the greenhouse results, five fungal isolates that showed high activity in improving plant growth were chosen for molecular identification using ITS rRNA sequence analysis. BLAST searches revealed their identities as members of five different genera (*Aspergillus*, *Botryotrichum*, *Chaetosphaeronema*, *Fusarium*, and *Penicillium*), all of them belonging to the phylum Ascomycota, which includes three classes, Eurotiomycetes, Sordariomycetes, and Dothideomycetes. A total of 21 sequences of close relatives were downloaded from the National Center for Biotechnology Information and combined with sequences in this study for phylogenetic tree construction (Figure 8). Based on the ITS phylogenetic tree, isolate NGB-WFS1 had an ITS sequence similarity of 99.5% to *Botryotrichim atrogriseum* CBS 130.28, and therefore it was classified in the genus *Botryotrichum*. Isolate NGB-WFS3 was closely related to reference strains corresponding to diverse species of *Penicillium* (ANI: 99.9%, BT: 99%), and therefore it was assigned to the genus *Penicillium*. Isolate NGB-WFS5 showed 99.8% ITS sequence similarity to *Aspergillus fumigatiaffinis* CBS 117186 and *A. fumigatiaffinis* CMV001G (the latter was isolated from soil in South Africa [3]), and thus it was identified as *Aspergillus*. Isolate NGB-WFS7 shared a 99.5% ITS sequence identity to the endophytic *Chaetosphaeronema* sp. SCE-N-O13, which was isolated from *Nepeta septemcrenata* growing in SKP in the South Sinai Governorate of Egypt [58], and therefore it was classified as *Chaetosphaeronema*. Finally, the endophyte NGB-WFE16 had 99.8% ITS sequence similarity with the soil fungus *Fusarium petersiae* CBS 143231, and consequently, it was defined as a *Fusarium*.

## 4. Discussion

PGPFs play important roles in the productivity of many crop plants by promoting plant growth and other activities such as protecting crops from diseases and compensating for the use of chemical fertilizers [59,60]. Our study aimed to investigate many PGP traits of both endophytic and rhizospheric fungi isolated from wild medicinal plants from the SKP in the South Sinai Governorate. Results revealed many growth-promoting traits such as IAA and siderophore production, phosphate solubilization efficiency, seedling vigor index, and the effect of fungi on the percentage of germination. The ability of fungal isolates to inhibit fungal pathogens and produce hydrolytic enzymes so that isolates can act as biocontrol agents, and the effect of fungal inoculation on wheat plant growth, were tested via a pot experiment.

IAA production by fungi enhanced lateral root formation and root hairs, thus increasing the nutrient absorption capacity of plants [61]. IAA played an important role in plant-microbe interactions, and thus it acted as a signaling molecule because it could affect gene expression in those microorganisms [62]. Also, fungal IAA supported the plants’ defenses against many phytopathogens [63].

In this study, all fungal isolates were able to produce IAA in the absence or presence of tryptophan in a culture medium. The NGB-WFS14 isolate produced the highest level of IAA (119.1 µg ml^−1^) in malt extract medium supplemented with 100 mg tryptophan L^−1^. This result was higher than that obtained by [64], who obtained a maximum IAA of 35 µg ml^−1^ produced by the endophytic *Aspergillus awamori* at 1000 mg tryptophan L^−1^.

Also, the NGB-WFS14 isolate showed an IAA level higher than reported by [65], who showed that *Trichoderma harzianum* produced an IAA level of between 13.4 and 24.3 µg ml^−1^ on a tryptic soy broth medium. IAA production is tryptophan dependent, and increasing its concentration in a broth medium increased the IAA levels synthesized by fungal isolates [66]

Our results were in agreement with the principle that IAA synthesis is increased by increased tryptophan concentrations [67]. This study revealed that our fungal isolates increased when tryptophan was increased at 100 mg L^−1^.

The finding that the endophytic isolate NGB-WFE16 synthesized 34.4 µg ml^−1^ in the presence or absence of tryptophan in a medium was in line with the results of [13], who hypothesized that endophytes had a unique capacity for IAA production without tryptophan. It reflects its natural habitat inside plant tissues where IAA could be synthesized free of tryptophan.

Phosphorus is considered one of the limiting factors for plant growth and crop production. Although it is abundant in agricultural soils, most of it is in an insoluble form, so it is unavailable for plant uptake in its bound form. Plants require 30 μmol l^−1^ of phosphorus for maximum crop production, but only 1 μmol l^−1^ is available in different soils [68,69]. Therefore, it is necessary to apply phosphate-solubilizing fungi to improve soil properties and enhance phosphorus availability for plants. Regarding the phosphate solubilization efficiency of fungal isolates in the present study, the highest amount of solubilized phosphorus was 181 µg mL^−1^ in the NGB-WFS2 isolate; that amount was higher than the amount obtained by [70], who found 83.42 ± 3.41 µg/mL to be the highest amount of phosphorus solubilized by *Penicillium daleae*. Our results were in line with the earlier studies of [70,71], who stated that increased solubilized phosphorus was accomplished by a decline in the pH of a medium. This was based on their finding of a sharp decline in pH (2.8, or highly acidic) in an NGB-WFS7 culture, which was correlated with a high solubilized phosphorus of 103 µg/mL.

Interestingly, not all high phosphate solubilization was accomplished with an acidic pH of a medium like that of the NGB-WFS5 isolate, which solubilized an amount of 147.7 µg/mL in a slightly alkaline medium at pH 8.6. This result was in agreement with that reported by [72], who suggested that phosphate solubilization might be dependent on mechanisms other than organic acid production.

Regarding the siderophores produced by fungi, siderophores known as low-molecular-weight iron-chelating agents could form complexes with iron that make it soluble and available to plants and unavailable to plant pathogens. This restricts phytopathogen growth [73]. In this study, the fungal isolates that showed the most siderophore units, 96.5% and 75.4%, were the endophytic isolates NGB-WFE16 and NGB-WFE15, respectively. These results were higher than those obtained by [74], who reported that the well-known antagonistic *Trichoderma* isolates had 85% and 65% siderophore-producing units. They are also linked to our finding that NGB-WFE16 and NGB-WFE15 had high antagonistic activity against *A. alternata,* with inhibition percentages of 76.2% and 57.9%, respectively.

The ability of fungal isolates to produce hydrolytic enzymes had indirect mechanisms for plant growth promotion [75]. In this study, most of the fungal isolates demonstrated good activity of hydrolytic enzymes. Results revealed that NGB-WFS2, NGB-WFS1, and NGB-WFE16 had the maximum cellulase, pectinase, and xylanase, with EIs of 2.1, 1.8, and 2.1, respectively. These results were in line with the principles of [76], who reported that these enzymes were responsible for facilitating the penetration of plant tissues by the fungal isolates and generating a symbiotic relationship with plants. In addition, these enzymes enhanced plant nutrition [77]. Regarding the chitinase activity of fungal isolates, NGB-WFS10 and NGB-WFS11 showed a high EI of 3.9 and 3, respectively. These results were in line with those of [78], who stated that the chitinase activity of fungal isolates could play a role in plant growth promotion by an indirect mechanism. The mechanism was the hydrolysis of chitin in the cell walls of phytopathogenic fungi. This finding, in turn, supported the idea of using these PGPFs in the biocontrol of fungal diseases.

One of the most important parameters was exploring the effect of both fungal spore suspension and the fungal filtrate on the wheat germination percentage and vigor index. They were considered good reflections of the ability of fungal isolates to be used as PGP agents. This study revealed that under in vitro conditions, a significantly higher germination percentage and improved seedling vigor were observed in grains treated with the fungal filtrates of NGB-WFS5, NGB-WFS6, and NGB-WFE16; the findings were 87% (1500), 94% (1651), and 96.5% (1767), respectively, compared with the control. These results were in agreement with the principles of [79], who reported that the seedling vigor and grain germination rate increased when grains of sorghum were treated with *Trichoderma asperellum*, *Epicoccum nigrum*, and *Amanita longipes.* They were linked to many PGP traits of these endophytes, such as the ability to produce IAA, which enhanced germination and root and shoot growth, resulting in improved seedling vigor compared with the control. Also, our results were in line with those of [51], who showed that the *Penicillium* filtrate could enhance the germination of wheat grains.

The improved seedling vigor is influenced by the PGP traits of endophytes, such as IAA production, which stimulates germination and root and shoot growth, whereas siderophore production and phosphate solubilization increase the sustained availability of micronutrients and phosphorus [80,81].

A pot experiment under greenhouse conditions was conducted to detect whether fungal isolates with at least one PGP trait could alleviate deficient fertilization. We also wanted to explore the ability of this isolate to promote plant growth.

Regarding the effect of fungal isolates on plant length, results showed that there was no significant difference between inoculated plants and the controls with 50% N and 100% N. At the same time, complete fertilization increased the plant length by 14.8%, whereas the fungal isolates had a maximum increase of 24.1% compared with the control at 50% N.

Fungal isolates have positive effects on the growth and yields of plants. The fungal isolates NGB-WFS1 and NGB-WFS5 increased the fresh shoot biomass of inoculated plants by 90% and 93%, respectively, and this exceeded the percentage that resulted from the non-inoculated control at a full dose (100% N). These results may be correlated with the ability of this isolate to produce IAA and solubilize phosphorus from soil, which could have a positive effect on the plant biomass, as observed. These results are in good agreement with those of [82], who reported that the production of IAA by endophytic *Alternaria* could enhance the growth parameters of wheat plants under drought conditions.

Interestingly, our results showed that the NGB-WFE17 isolate increased the root fresh biomass by 135% compared with the control at 50% N. This large increase in root biomass was due to the fact that that endophytic isolate could produce IAA in the presence or absence of tryptophan. Our results were in agreement with those of [80], who found that endophytic bacteria with more than one PGP trait could help raise the plant growth indices more than rhizospheric bacteria.

Regarding the dry shoot biomass, our results revealed that the control with a full dose of N fertilizer (100% N) could increase the shoot dry biomass by 31% compared with the control at 50% N. At the same time, inoculation with NGB-WFS5 increased the shoot dry biomass by 97% compared with the control at 50% N. This may have been due to the high amount of phosphorus solubilized (147 µg mL^−1^) in Pikovskaya’s culture medium observed for this isolate. Our results were in line with the observations of [81], who reported that the shoot dry biomass of maize plants increased by 62% compared with the control through inoculation by the phosphate-solubilizing *Penicillium* sp.

This study observed clearly that endophytic fungi play a crucial role in promoting the growth of wheat plants through many PGP traits. Our results revealed that the N content of wheat shoots increased by 68% in plants inoculated with NGB-WFE16 (*Fusarium petersiae*) compared with the non-inoculated control at 50% N. The percentages exceeded the increase caused by fertilization with N chemical fertilizers. Our results were in agreement with those of [79], who reported that wheat plants inoculated with endophytic *A. alternata* (LQ1230) acquired an accumulation of carbon and nitrogen. This observation was correlated with the ability of this isolate to increase the shoot dry biomass, in the same principle with [83], who concluded that dark septate fungi like *Fusarium petersiae* could increase nitrogen content through increased nitrogen availability for host plants, not through direct absorption of nitrogen.

Also, many physiological processes were affected by inoculation with PGPFs [79]. Our results revealed that plants inoculated with fungal isolates NGB-WFE15 (*Penicillium chrysogenum*), NGB-WFE16 (*Fusarium petersiae*), and NGB-WFS18 (*Trichoderma atroviride*) had high total chlorophyll contents of 3.1, 3.0, and 3.0 mg g^−1^, respectively, compared with both of the non-inoculated controls (50% N, 100% N). These results were in line with those of [84], who reported that wheat plants inoculated with *Trichoderma reesei* could tolerate salt stress and showed a high level of total chlorophyll compared with non-inoculated wheat plants.

The determination of carotenoids is considered a vital parameter in determining the stress tolerance and scavenging ability of reactive oxygen species (ROS). Our results showed that plants inoculated with NGB-WFS1, NGB-WFE15, NGB-WFE17, and NGB-WFS18 had high levels of carotenoids (0.77, 0.65, 0.66, and 0.81 mg g^−1^, respectively) compared with the non-inoculated controls (50% N and 100% N). These results were in agreement with those of [85], who concluded that carotenoids might be decreased in the presence of stress.

Many authors have suggested different mechanisms of plant growth-promoting fungi (PGPF) for phytostimulation. The mechanism for producing acids is recorded for the first time in this study, where PGPF able to produce acids via PVK broth medium; this supported wheat plant soil with acidic pH, as isolates NGB-WFS1, NGB-WFS3, NGB-WFS7, NGB-WFE15, and NGB-WFE16 had PH (2.4, 6.6, 2.8, 1.9, and 6) accompanied with a high nitrogen content of wheat (12.9, 12.4, 12.1, 19.5 & 15.6 mg N/plant), respectively. Our results are in agreement with the finding of [86], who stated that soil acidification is effective in improving the N availability of soil; in addition, [87] reported that N availability and N uptake were significantly higher on pH-neutral soil than on more alkaline soil [86]. Other mechanisms included the production of hydrolytic enzymes, which are considered a crucial point for plant growth promotion. In our study, the wheat plants inoculated with PGPF isolates (NGB-WFS1, NGB-WFS3, NGB-WFS7, NGB-WFE15, and NGB-WFE16) showed high nitrogen content. This correlated with the ability of these isolates to produce many hydrolytic enzymes (mainly cellulose, pectinase, and xylanase) with different enzymatic indexes, as shown in Results Table 4, since [88], who reported that nitrogen assimilation could be accelerated in soil amended with barely infested grains which could produce cellulose and starch-decomposing enzymes, despite their ability for IAA or siderophore production. So, nutrient release by mineralization is considered an acceptable mechanism for plant growth promotion in fungi rather than mycorrhizae.

## 5. Conclusions

Despite being a decades-old technology, the use of fungal species as biological control agents has grown exponentially in recent years. Due to the growing concern about the environmental impacts of agricultural processes, as well as the search for healthier foods free of chemical compounds that are harmful to health, scientific research is progressing toward the exploitation of endophytic and rhizospheric microorganisms for this purpose. The ability of several fungal species, including those historically used for pest control, to colonize crops endophytically has proven to be a very promising mechanism for achieving the desired sustainability in agriculture. Endophytic fungi are beneficial because they provide several direct and indirect benefits to crop plants, as stated in this research. It is reasonable to assume that no synthetic molecule can provide such a wide range of positive interactions as these microorganisms. Thus, the use of endophytic fungi proves to be a promising alternative in the fields of biocontrol, biostimulation, and biofertilization, demonstrating that such organisms are a valuable resource for research and business.

## Figures and Tables

**Table 1:** Sites of collection and host plants.

Longitude	Latitude	Site of Collection	Plant Family	Host Plant
28.309 N	33.984 E	Wadi Tarafa	Labiatae	*Cleome droserifolia* (Forssk.) Delile
28.539 N	33.979 E	Shaq Elgragnia	Labiatae	*Thymus bovei* Benth.
28.542 N	33.964 E	WadiElArba’een	Labiatae	*Marrubium alysson* L.
33.934 E	28.553 N	Shaq Itlah	Compositae	*Sonchus oleraceus* L.
28.568 N	33.929 E	Wadi Eltalaa	Zygophyllaceae	*Peganum harmala* L.
33.934 E	28.553 N	Shaq Itlah	Cruciferae	*Diplotaxis harra* (Forssk.) Boiss.
34.0148 E	28.550 N	Wadi Sdod	Adianteacea	*Cheilanthes vellea* (Aiton) F. Muell.
28.539 N	33.977 E	Gebel Musa	Compositae	*Conyza stricta* Willd.
33.940 N	28.538 E	Elfaraa	Caryophyllaceae	*Silene schimperiana* Boiss.

**Table 2:** Endophytic and rhizospheric taxa hosted by different plant species under investigation.

Isolates Code	Host Plant	Phenotypic Identification	Molecular Confirmation	Accession No.
NGB-WFS1 *	*Cleome droserifolia* (Forssk.) Delile	*Botryotrichum atrogriseum* J.F.H. Beyma	*Botryotrichum atrogriseum*	LC642736
NGB-WFS2	*Thymus bovei* Benth.	*Penicillium chrysogenum* Thom	N/A	N/A
NGB-WFS3 *	*Thymus bovei* Benth.	*Penicillium chrysogenum* Thom	*Penicillium*	LC642737
NGB-WFS4	*Marrubium alysson* L.	*Chaetosphaeronema achilleae* S.K. Huang & K.D. Hyde	N/A	N/A
NGB-WFS5	*Sonchus oleraceus* L.	*Aspergillus fumigatiaffinis* S.B. Hong, Frisvad & Samson	*Aspergillus fumigatiaffinis*	LC642738
NGB-WFS6	*Peganum harmala* L.	*Alternaria alternata* (Preuss) Woudenb. & Crous	N/A	N/A
NGB-WFS7 *	*Peganum harmala* L.	*Chaetosphaeronema achilleae* S.K. Huang & K.D. Hyde	*Chaetosphaeronema* sp.	LC642739
NGB-WFS8	*Peganum harmala* L.	*Acrophialophora levis* Samson & T. Mahmood	N/A	N/A
NGB-WFS9	*Peganum harmala* L.	*Aspergillus versicolor* (Vuill.) Tirab.	N/A	N/A
NGB-WFS10	*Diplotaxis harra* (Forssk.) Boiss.	Sterile mycelium	N/A	N/A
NGB-WFS11	*Sonchus oleraceus* L.	*Geotrichum* sp.	N/A	N/A
NGB-WFS12	*Sonchus oleraceus* L.	*Penicillium chrysogenum* Thom	N/A	N/A
NGB-WFS13	*Diplotaxis harra* (Forssk.) Boiss.	*Aspergillus versicolor* (Vuill.) Tirab.	N/A	N/A
NGB-WFS14	*Diplotaxis harra* (Forssk.) Boiss.	*Chaetosphaeronema achilleae* S.K. Huang & K.D. Hyde	N/A	N/A
NGB-WFE15	*Cheilanthes vellea* (Aiton) F.Muell	*Penicillium chrysogenum* Thom	N/A	N/A
NGB-WFE16 *	*Conyza stricta* Willd.	*Fusarium oxysporum* Schltdl.	*Fusarium petersiae*	LC642740
NGB-WFE17	*Silene schimperiana* Boiss.	*Alternaria botrytis* (Preuss) Woudenb. & Crous	N/A	N/A
NGB-WFS18	*Diplotaxis harra* (Forssk.) Boiss.	*Trichoderma atroviride* P. Karst.	N/A	N/A

Where WFS: Rhizospheric isolate; WFE: Endophytic isolate. * denotes to isolates confirmed by molecular identification.

**Table 3:** Percentage growth inhibition against *Alternaria alternata.* Percent siderophore production of tested fungal isolates.

Isolates	Pathogen Growth Inhibition %			Siderophore Production (% SU)		
NGB-WFS1	65.9	±	0.8 ^b–e^	25.9	±	0.10 ^h^
NGB-WFS2	59.5	±	2.4 ^e,f^	3.1	±	0.05 ^n^
NGB-WFS3	63.5	±	7.9 ^c–f^	19.3	±	0.05 ^i^
NGB-WFS4	69.1	±	0.8 ^b–e^	33.7	±	0.05 ^g^
NGB-WFS5	73.8	±	4.0 ^b–d^	1.5	±	0.10 ^o^
NGB-WFS6	64.3	±	0.8 ^b–f^	33.6	±	0.55 ^g^
NGB-WFS7	62.7	±	0.8 ^d–f^	23.0	±	0.25 ^i^
NGB-WFS8	77.0	±	0.8 ^a,b^	0.0	±	0.00 ^p^
NGB-WFS9	56.4	±	0.8 ^e,f^	44.7	±	0.15 ^e^
NGB-WFS10	59.6	±	5.6 ^e,f^	61.7	±	0.05 ^c^
NGB-WFS11	59.5	±	0.8 ^e,f^	57.1	±	1.85 ^d^
NGB-WFS12	58.0	±	4.0 ^e,f^	16.8	±	0.10 ^l^
NGB-WFS13	55.6	±	4.8 ^e,f^	36.2	±	0.30 ^f^
NGB-WFS14	52.4	±	4.8 ^f^	17.3	±	0.30 ^k^
NGB-WFE15	57.9	±	0.8 ^e,f^	75.4	±	0.14 ^b^
NGB-WFE16	76.2	±	9.5 ^a–c^	96.5	±	0.43 ^a^
NGB-WFE17	63.5	±	1.6 ^c–f^	14.6	±	0.32 ^m^
NGB-WFS18	87.3	±	1.6 ^a^	44.4	±	0.09 ^e^

Mean values followed by the same letters (a, b, c, etc.) are not significantly different according to Duncan’s multiple range test at *p* ≤ 0.05.

**Table 4:** Enzymatic index of hydrolytic enzymes of fungal isolates.

Isolate Code	Enzymatic Index			
	Xylanase	Chitinase	Xylanase	Chitinase
NGB-WFS1	1.4 ^b,c^	1.8 ^a^	1.1 ^b–d^	1.3 ^d,e^
NGB-WFS2	2.0 ^a^	1.5 ^a,b^	2.0 ^a,b^	1.2 ^d,e^
NGB-WFS3	1.6 ^b^	1.6 ^a,b^	1.4 ^a–d^	1.2 ^d,e^
NGB-WFS4	1.0 ^d^	1.0 ^a–c^	0.0 ^e^	1.0 ^e^
NGB-WFS5	1.0 ^d^	1.0 ^a–c^	1.0 ^c,d^	1.1 ^d,e^
NGB-WFS6	1.1 ^d^	0.0 ^d^	0.0 ^e^	1.0 ^e^
NGB-WFS7	1.4 ^b,c^	1.3 ^a–c^	1.3 ^a–d^	1.4 ^d^
NGB-WFS8	1.1 ^d^	1.0 ^a–c^	0.0 ^e^	1.0 ^e^
NGB-WFS9	1.6 ^b^	1.3 ^a–c^	1.4 ^a–d^	1.8 ^c^
NGB-WFS10	1.0 ^d^	1.1 ^a–c^	0.5 ^d,e^	3.9 ^a^
NGB-WFS11	1.1 ^d^	1.8 ^a^	1.3 ^a–d^	3.0 ^b^
NGB-WFS12	1.6 ^b^	1.4 ^a–c^	1.5 ^a–c^	1.3 ^d,e^
NGB-WFS13	1.3 ^c,d^	1.0 ^b,c^	1.3 ^a–d^	1.4 ^d^
NGB-WFS14	1.5 ^b,c^	1.3 ^a–c^	1.4 ^a–d^	1.0 ^e^
NGB-WFE15	1.1 ^d^	1.7 ^a,b^	1.1 ^b–d^	1.1 ^d,e^
NGB-WFE16	1.3 ^c,d^	0.7 ^c,d^	2.1 ^a^	1.0 ^e^
NGB-WFE17	1.0 ^d^	1.5 ^a,b^	1.1 ^b–d^	1.2 ^d,e^
NGB-WFS18	1.0 ^d^	1.0 ^a–c^	1.9 ^a–c^	1.0 ^e^

Mean values followed by the same letters (a, b, c, etc.) are not significantly different according to Duncan’s multiple range test at *p* ≤ 0.05.

**Table 5:** Influence of culture filtrate and spore suspension of fungal isolates on the percent of grain germination and seedling vigor of wheat grains.

Isolate Code	Percent of Grain Germination (%)		Seedling Vigor	
	Fungi Filterate	Fungi Spore Suspension	Fungi Filterate	Fungi Spore Suspension
NGB-WFS1	90.5 ^a^	79.5 ^a–c^	1236 ^a–e^	1304 ^a,b^
NGB-WFS2	81.5 ^a,b^	50.5 ^d^	1362 ^a–d^	871 ^b,c^
NGB-WFS3	78 ^a,b^	75 ^a–d^	1238 ^a–e^	1270 ^a,b^
NGB-WFS4	77 ^a,b^	80 ^a–c^	872 ^d–f^	1043 ^a–c^
NGB-WFS5	87 ^a^	70 ^a–d^	1500 ^a,b^	1147 ^a–c^
NGB-WFS6	94 ^a^	81 ^a–c^	1651 ^a,b^	1308 ^a,b^
NGB-WFS7	53.5 ^a,b^	61.5 ^b–d^	603 ^f^	727 ^c^
NGB-WFS8	86 ^a^	0.00 ^e^	1482 ^a–c^	0 ^d^
NGB-WFS9	72 ^a,b^	81.5 ^a,b^	1327 ^a–d^	1362 ^a,b^
NGB-WFS10	69.5 ^a,b^	70.5 ^a–d^	1168 ^b–e^	1276 ^a,b^
NGB-WFS11	76.5 ^a,b^	90.5 ^a^	1385 ^a–d^	1533 ^a^
NGB-WFS12	73.5 ^a,b^	68.5 ^a–d^	1287 ^a–e^	1178 ^a–c^
NGB-WFS13	67 ^a,b^	71 ^a–d^	1200 ^a–e^	1199 ^a–c^
NGB-WFS14	76 ^a,b^	0.00 ^e^	729 ^e,f^	0 ^d^
NGB-WFE15	66.5 ^a,b^	61.5 ^b–d^	1131 ^b–f^	1081 ^a–c^
NGB-WFE16	96.5 ^a^	53 ^c,d^	1767 ^a^	883 ^b,c^
NGB-WFE17	78.5 ^a,b^	74 ^a–d^	1339 ^a–d^	1200 ^a–c^
NGB-WFS18	77 ^a,b^	68.5 ^a–d^	1402 ^a–d^	887 ^b,c^
Control	73.5 ^a,b^	70.5 ^a–d^	910 ^c–f^	888 ^b,c^

Mean values followed by the same letters (a, b, c, etc.) are not significantly different according to Duncan’s multiple range test at *p* ≤ 0.05.

**Table 6:** Physical and chemical properties of sandy soil used in the plant inoculation assay.

Property	Value
Particle size distribution (%)	
Sand	90.1
Silt	3.9
Clay	6.0
Texture grade	Sandy
CaCo_3_ (%)	1.61
Saturation percent S.P (%)	21.50
pH	7.82
E.C. (dS m^−1^ at 25 °C)	0.32
Soluble cations (meq/L)	
Ca^2+^	0.54
Mg^2+^	0.33
Na^+^	1.62
K^+^	0.65
Soluble anions (meq/L)	
CO_3_^−2^	0.00
HCO_3_^−^	0.88
Cl^−^	0.59
SO_4_^−2^	1.67
Total N (%)	0.021
Total Soluble-N (mg kg^−1^)	16.30
Available-P (mg kg^−1^)	6.71
Available-K (mg kg^−1^)	52.10
Organic matter (%)	0.23
DTPA extractable (ppm)	
Fe	1.62
Mn	0.31
Zn	0.42
Cu	0.18

**Table 7:** Effect of fungal inoculation on photosynthetic pigments of wheat plants under pot trial.

Isolate	Chlorophyll a (mg g^−1^)			Chlorophyll b (mg g^−1^)			Total Chlorophyll (mg g^−1^)			Carotenoids (mg g^−1^)		
NGB-WFS1	2.0	±	0.07 ^a^	0.56	±	0.06 ^a,b^	2.6	±	0.13 ^a–d^	0.77	±	0.06 ^a,b^
NGB-WFS2	1.8	±	0.12 ^a–d^	0.43	±	0.06 ^b–d^	2.2	±	0.18 ^c–g^	0.63	±	0.07 ^b–e^
NGB-WFS3	1.7	±	0.00 ^a–e^	0.40	±	0.01 ^c,d^	2.1	±	0.01 ^d–g^	0.60	±	0.01 ^c–e^
NGB-WFS4	1.6	±	0.03 ^a–e^	0.38	±	0.00 ^c,d^	2.1	±	0.04 ^d–g^	0.58	±	0.02 ^c–e^
NGB-WFS5	1.7	±	0.02 ^a–e^	0.40	±	0.01 ^c,d^	2.1	±	0.03 ^d–g^	0.58	±	0.03 ^c–e^
NGB-WFS6	1.5	±	0.41 ^d,e^	0.34	±	0.12 ^d,e^	1.9	±	0.53 ^f,g^	0.54	±	0.14 ^d,e^
NGB-WFS7	1.5	±	0.08 ^c–e^	0.37	±	0.05 ^c,e^	1.9	±	0.14 ^f,g^	0.56	±	0.07 ^d,e^
NGB-WFS9	1.6	±	0.26 ^b–e^	0.38	±	0.07 ^c,d^	2.0	±	0.34 ^e–g^	0.59	±	0.04 ^c–e^
NGB-WFS10	1.7	±	0.21 ^a–e^	0.40	±	0.09 ^c,d^	2.1	±	0.31 ^d–g^	0.58	±	0.10 ^c–e^
NGB-WFS11	1.8	±	0.02 ^a–d^	0.45	±	0.00 ^b–d^	2.3	±	0.02 ^c–f^	0.66	±	0.02 ^b–d^
NGB-WFS12	1.6	±	0.21 ^a–e^	0.37	±	0.07 ^c–e^	2.0	±	0.30 ^e–g^	0.59	±	0.05 ^c–e^
NGB-WFS13	1.7	±	0.00 ^a–e^	0.40	±	0.00 ^c,d^	2.1	±	0.01 ^d–g^	0.62	±	0.04 ^c–e^
NGB-WFE15	1.9	±	0.02 ^a–c^	0.43	±	0.04 ^b–d^	3.1	±	0.00 ^a^	0.65	±	0.03 ^b–d^
NGB-WFE16	1.8	±	0.05 ^a–d^	0.34	±	0.04 ^d,e^	3.0	±	0.11 ^a,b^	0.59	±	0.02 ^c–e^
NGB-WFE17	1.9	±	0.01 ^a–c^	0.45	±	0.02 ^b–d^	2.7	±	0.13 ^a–c^	0.66	±	0.01 ^b–d^
NGB-WFS18	2.0	±	0.00 ^a,b^	0.66	±	0.02 ^a^	3.0	±	0.05 ^a,b^	0.81	±	0.01 ^a^
Control (0 N)	1.1	±	0.28 ^f^	0.23	±	0.08 ^e^	1.3	±	0.37 ^h^	0.49	±	0.00 ^e^
Control (50 N)	1.8	±	0.27 ^a–e^	0.42	±	0.06 ^a,b^	2.2	±	0.35 ^c–g^	0.63	±	0.12 ^b–e^
Control (100 N)	2.0	±	0.21 ^a,b^	0.52	±	0.06 ^b–d^	2.5	±	0.39 ^b–e^	0.72	±	0.05 ^a–c^

Mean values followed by the same letters (a, b, c, etc.) are not significantly different according to Duncan’s multiple range (*p* ≤ 0.05).

## Data Availability

Not applicable.
